# A retrospective study on the reasons for second molar extraction

**DOI:** 10.3389/fdmed.2025.1627563

**Published:** 2025-11-10

**Authors:** Chengyi Wang, BaoCheng Yao, Shiyu Qiu, Shiqi Zou, Yu Wu, Xiao Wang

**Affiliations:** Dental Department, Peking University Third Hospital, Peking, China

**Keywords:** second molar extraction, epidemiological study, impacted third molar teeth, cone-beam computed tomography, retrospective study

## Abstract

**Objective:**

The reasons for second molar extraction include endodontics, periodontal disease, and impacted third molars. This retrospective study analyzed medical records and image examinations of patients who underwent second molar extraction to clarify the proportional distribution of different causes.

**Materials and methods:**

Medical records and imaging data of patients who underwent second molar extraction between January 1, 2020 and December 31, 2022 were systematically collected. Two senior dentists analyzed and recorded patient demographics, clinical diagnoses, and extraction causes. Inter-rater consistency was verified using Kappa testing.

**Results:**

A total of 1,818 valid cases were included with a mean age of 52.9 years (male: 864 cases; female: 954 cases). Endodontic factors accounted for 1,045 s molar extractions (57.43%) and periodontal factors contributed to 588 extractions (32.40%), both showing significant age-related correlations (*p* < 0.05). Impacted third molar factors led to 185 extractions (10.01%) with no significant age-dependent association (*p* > 0.05). Impacted third molars contributed to 13.97% of mandibular second molar extractions, while only 5.73% to maxillary second molar extractions.

**Conclusions:**

The main reasons for extracting second molars were endodontic factors, followed by periodontal factors, and impacted third molars. Advanced age was significantly associated with increased extraction risks attributable to both endodontic and periodontal disease. Impacted third molar-related factors accounted for a larger proportion in cases of mandibular second molar extractions.

## 1 Introduction

The second molar plays a critical role in mastication. Sato et al. demonstrated that second molars account for 20%–30% of the total occlusal contact area in adults, contributing 10%–15% to chewing efficiency. Unilateral loss of second molars reduces the overall efficiency by approximately 25% (1, 2). Additionally, second molar eruption coincides with peak jawbone development, providing mechanical stimulation to enhance alveolar bone height (3). Its position at the dental arch terminus helps maintain the posterior arch width and occlusal vertical dimension, preventing collapse (4). An increasing volume of evidence indicates that there is a bidirectional relationship between tooth loss and systemic diseases, including cardiovascular and cerebrovascular diseases, diabetes, hypertension, and even cancer (5–7).

Compared to other teeth, second molars can be extracted due to external root resorption or caries due to impacted third molars (2–4, 8, 9). It is notable that impacted third molars can occasionally exert adverse impacts on the dental tissues of adjacent second molars and the surrounding alveolar bone. Extraction of second molars can be performed due to external root resorption or severe caries caused by impacted third molars. Currently, a unified consensus has not been reached on whether impacted third molars should be prophylactically extracted or not (10). Additionally, prior to the onset of clinical symptoms such as pain or swelling, patients generally tend to choose conservative observation for impacted third molars.

Cone-beam computed tomography (CBCT), with its high spatial resolution, low radiation dose, and 3D imaging capabilities, has become indispensable in diagnosing dental and maxillofacial pathologies (11). The imaging examination data employed in this study primarily stemmed from CBCT data. By integrating patients' prior medical records with CBCT images, we were able to precisely identify the reasons for the extraction of second molars.

Epidemiological studies on the extraction of second molars are scarce, and the potential adverse effects of impacted third molars on the second molar may have been underestimated. This study aimed to analyze the reasons for the extraction of second molars. To clarify the adverse effects of impacted third molars to second molars, which were thought to be underestimated.

## 2 Materials and methods

A systematic retrospective review was conducted on patients undergoing second molar extraction in the Department of Stomatology, Peking University Third Hospital, between January 1, 2020 and December 31, 2022. Demographic parameters (name, ID, sex, age) and clinical documentation [visit dates, electronic health records (EHR), CBCT imaging] were collected. The voxel size of the CBCT instrument we used (LARGEV, Smart3D-X, China) was 0.05–0.25 mm, the field of view was 15 cm × 10 cm/8 cm × 8 cm/5 cm × 8 cm, the tube voltage setting was 60–100 KV, and the tube current settings were 2–10 mA. Ethical approval was obtained from Peking University Third Hospital (Ethics No: IRB00006761-M2024038).

Inclusion criteria:

A) Patients who had second molars extracted.
B) Entire medical records and imaging examination data were available.

 Exclusion criteria:

A) Extraction for orthodontic indications or prosthodontic purposes.

Following the acquisition of complete demographic data from all eligible patients, two experienced dentists analyzed the clinical profiles, independently. The reasons for second molar extractions were classified through the comprehensive evaluation of medical records and imaging examination data, with inter-examiner reliability assessments conducted to ensure diagnostic consistency. All image analyses were carried out using Kavo Exam Vision software (Kavo, Germany). When the two dentists had different diagnoses for the cause of tooth extraction, they discussed the data to determine the final cause based on the relevant information. If necessary, they also consulted a third experienced dentist.

**Figure 1:**
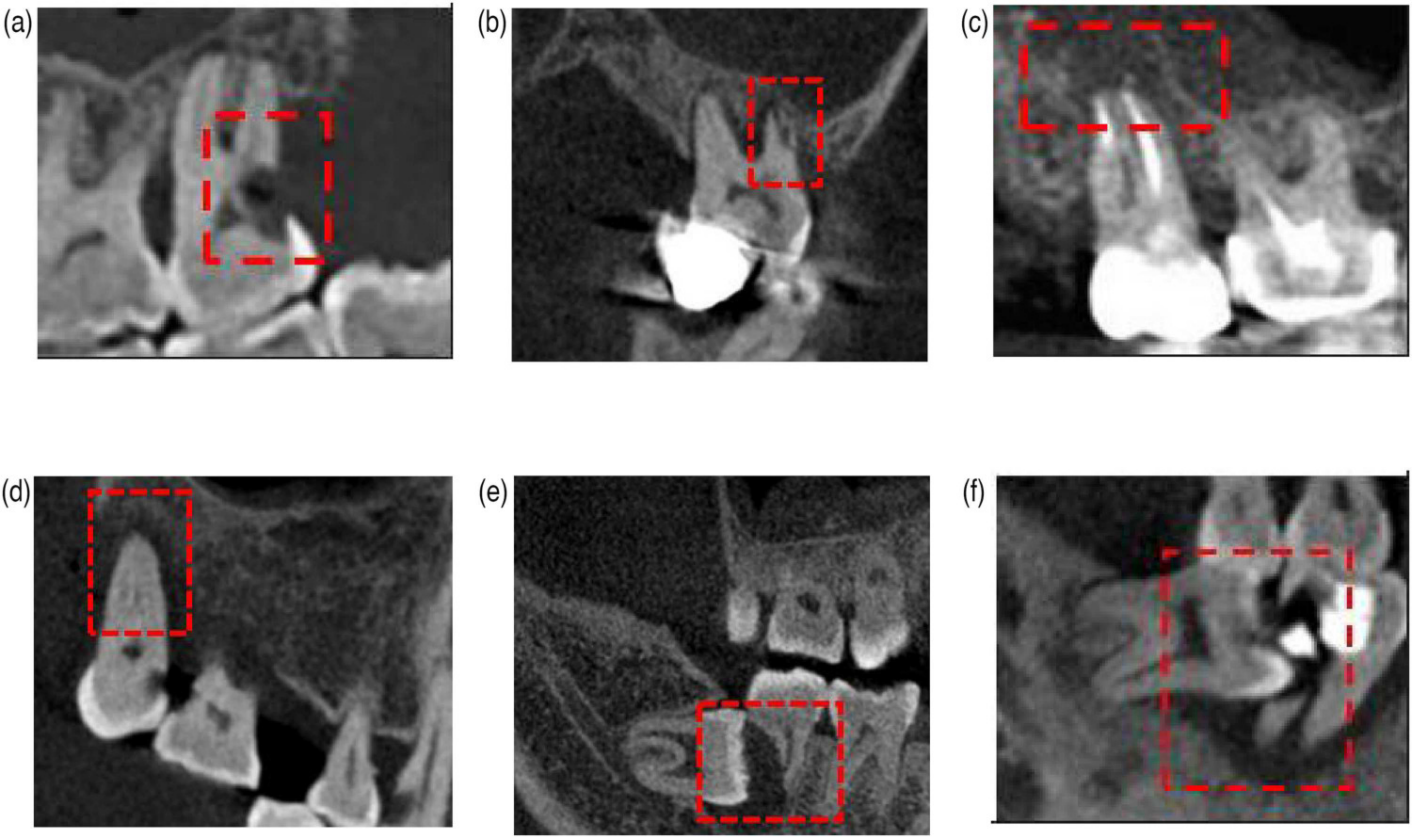
Reasons for second molar extractions. **(a)** Endodontic factors: severe caries. **(b)** Endodontic factors: cracked teeth. **(c)** Endodontic factors: periapical lesions. **(d)** Periodontal factors: severe periodontitis. **(e)** Impacted third molar-related factors. **(f)** Impacted third molar-related factors.

The causes of second molar extraction were categorized into three distinct groups: endodontic factors, periodontal factors, and impacted third molar-related factors (Table 1, Figure 1). Patient demographics and causes were analyzed using SPSS 26.0 (IBM, USA). A significance threshold of *P* < 0.05 was adopted for statistical inference.

**Table 1:** Etiology and classification criteria for second molar extraction.

Reasons for second molar extraction	Classification criteria
Endodontic factors	Severe caries, cracked tooth, periapical lesions, combined endodontic-periodontal lesions
Periodontal factors	Severe periodontitis
Impacted third molar-related factors	Severe external root resorption or alveolar bone resorption in the second molar caused by impacted third molars, severe distal caries caused by impacted third molar

## 3 Results

This study included 1,818 valid cases (954 females and 864 males) and the population ranging in age from 17 to 94 years, with a mean age of 52.92 ± 2.31 years. The inter-rater reliability for etiological classification between two clinicians demonstrated substantial agreement, with a Kappa coefficient of 0.82 (95% CI: 0.78–0.86).

In descending order, the number of second molars analyzed in the four different oral sections were mandibular right second molars (500 cases, 27.50%), mandibular left second molars (481 cases, 26.46%), maxillary right second molars (422 cases, 23.21%), and maxillary left second molars (415 cases, 22.83%). The maxillary/mandibular ratio was 0.85. The distribution of the reasons for extractions was as follows: endodontic factors, 57.43% (1,045 cases); periodontal factors, 32.40% (588 cases); and impacted third molar factors, 10.01% (185 cases).

**Figure 2:**
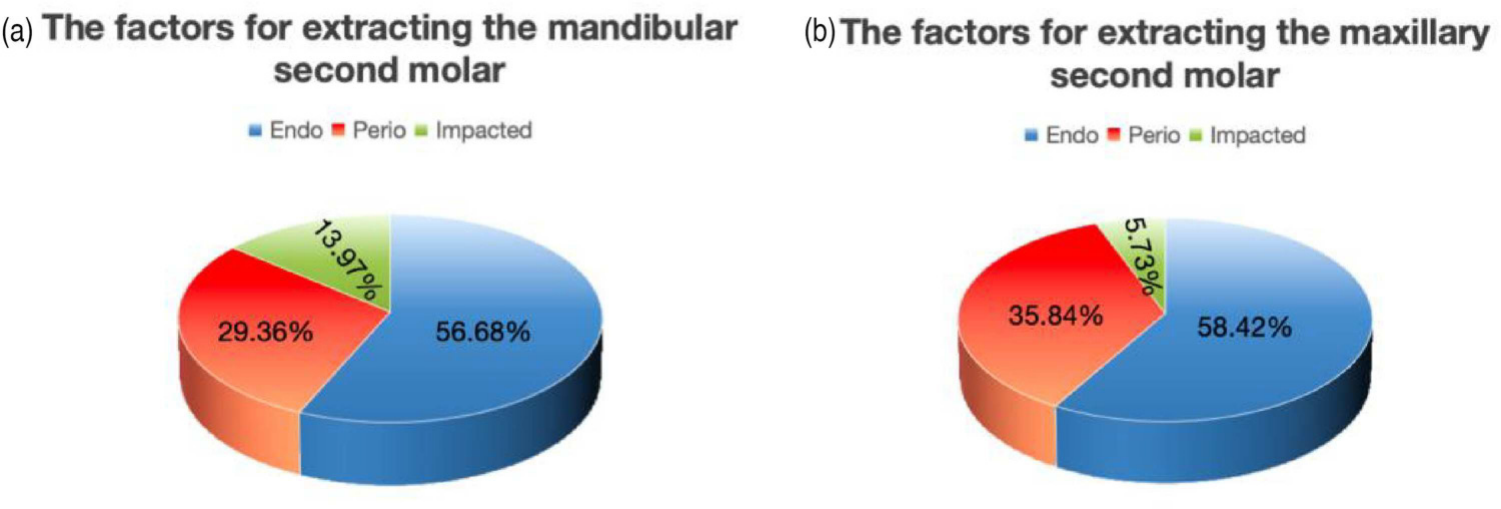
**(a)** The factors for extracting mandibular second molars. **(b)** The factors for extracting maxillary second molars.

More specifically, the distribution of the causes for mandibular second molar extractions was as follows: endodontic factors, 56.68% (556 cases); periodontal factors, 29.36% (288 cases); and impacted third molar factors, 13.97% (137 cases) (Figure 2A). The distribution of causes for maxillary second molar extractions was as follows: endodontic factors, 58.42% (489 cases); periodontal factors, 35.84% (300 cases); and impacted third molar factors, 5.73% (48 cases) (Figure 2B).

**Figure 3:**
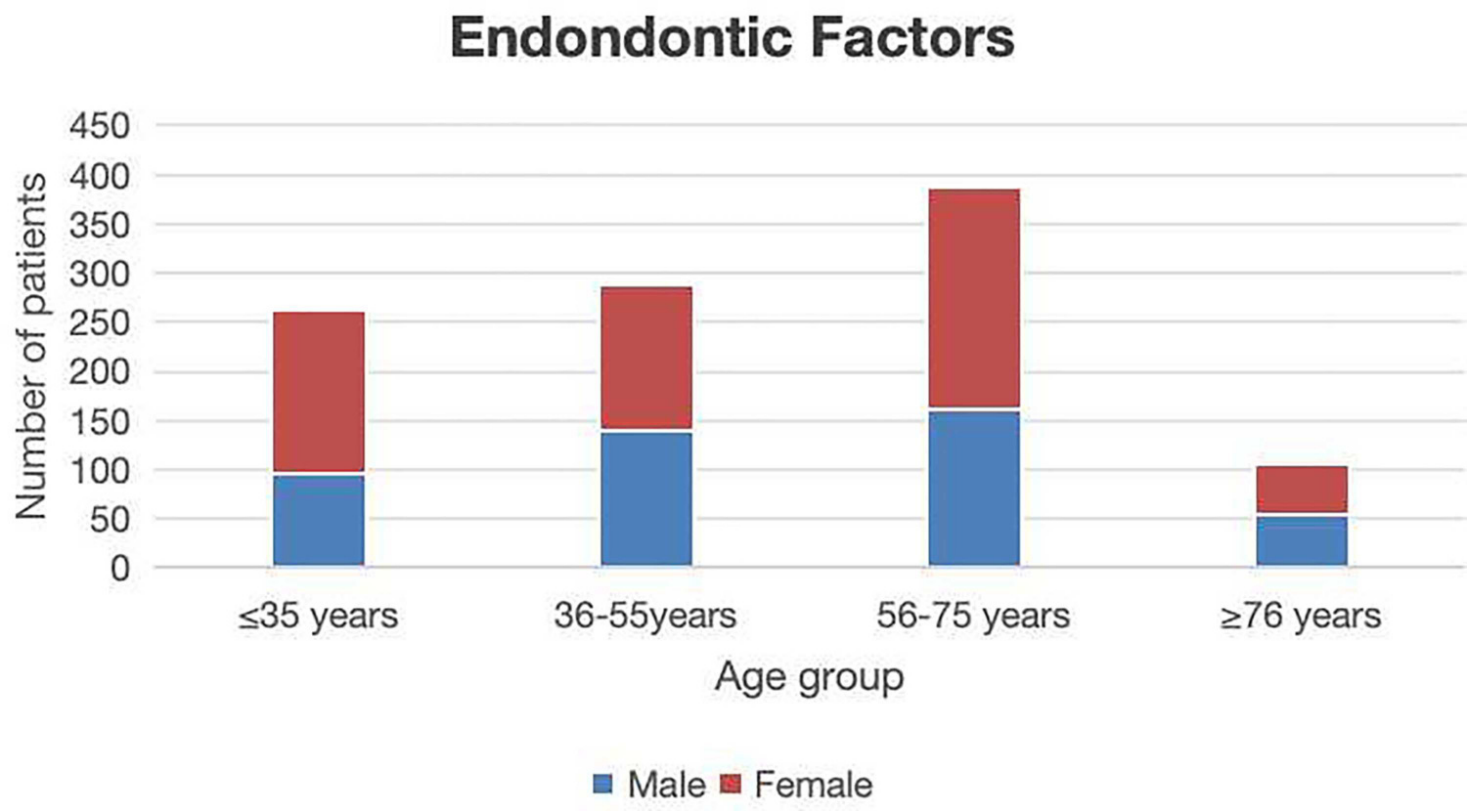
The sex and age distribution of second molar extractions due to endodontic factors.

**Figure 4:**
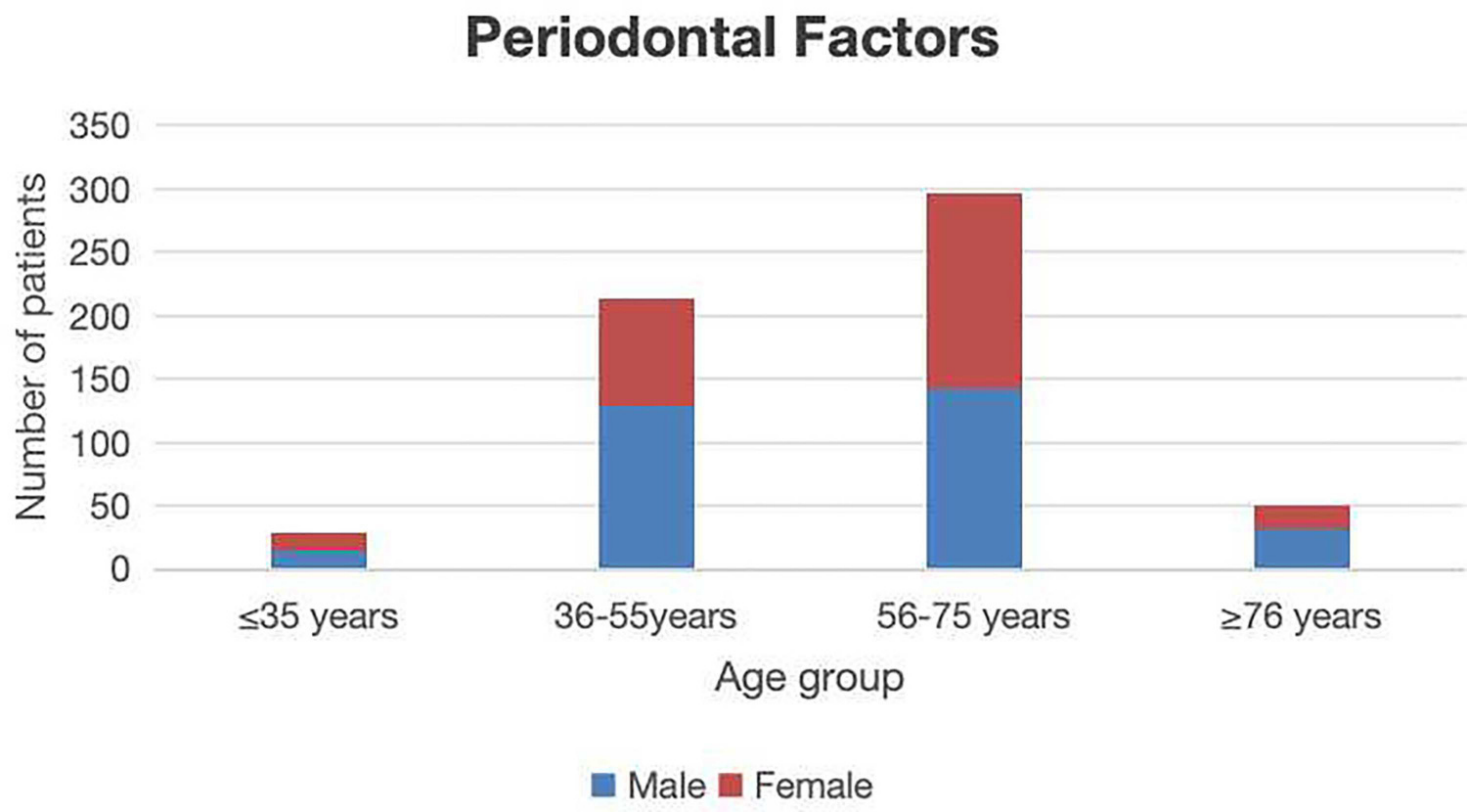
The sex and age distribution of second molar extractions due to periodontal factors.

**Figure 5:**
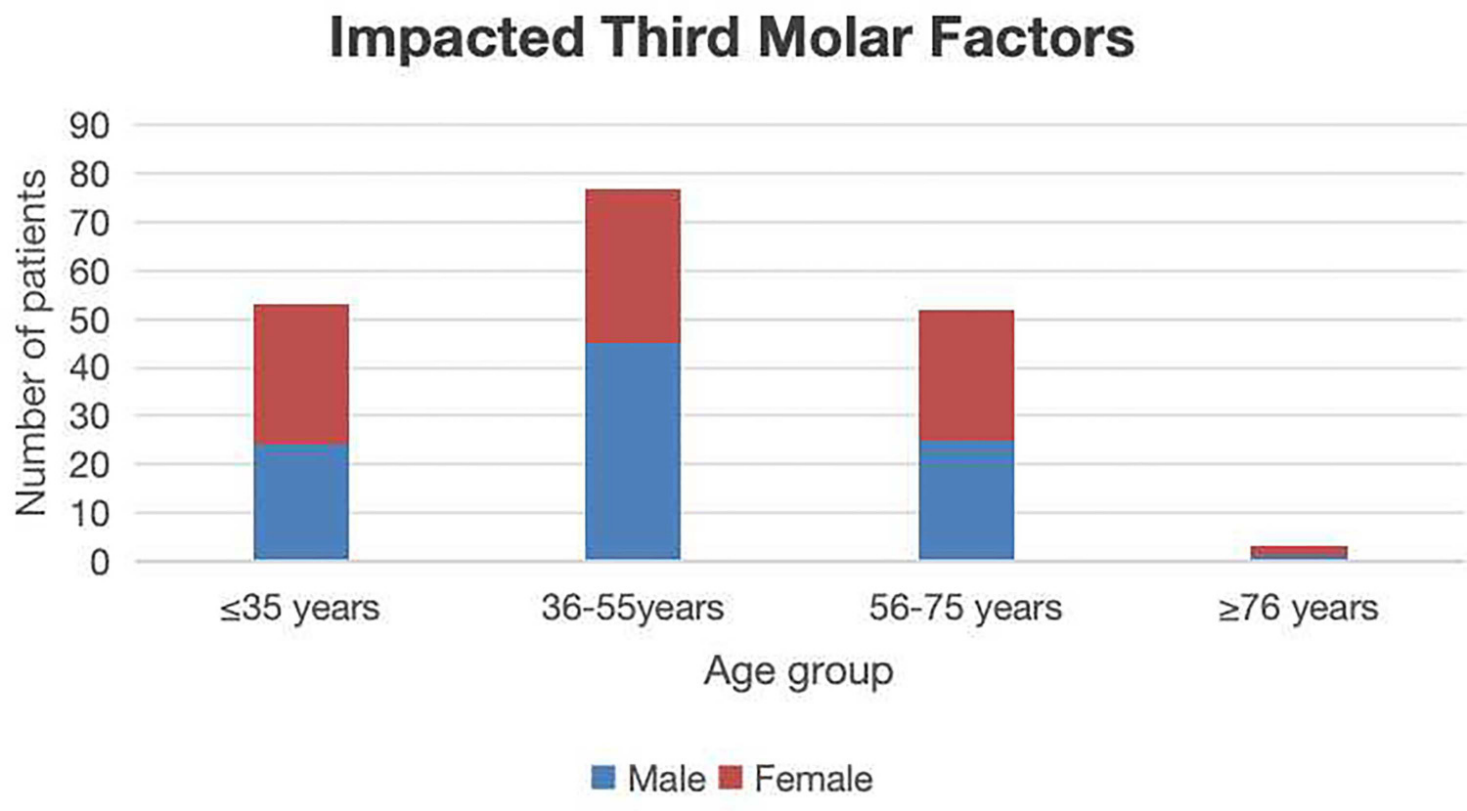
The sex and age distribution of second molar extractions due to impacted third molar factors.

All cases were divided into four groups, including a group <35 years of age (344 cases); 36–55 years of age (578 cases); 56–75 years of age (737 cases), and >76 years of age (159 cases). Statistically significant differences in patient distributions were observed across age groups (*P* < 0.05). The 56–75 year cohort represented the predominant age group for both endodontic disease and periodontal disease. In contrast, the 36–55 year group exhibited the highest frequency of extractions attributable to impacted third molars. Patients aged >76 years demonstrated the lowest extraction frequency (Table 2). Endodontic factors accounted for 1,045 s molar extractions showing a significantly positive correlation with advancing age (*p* < 0.05) (Figure 3). Periodontal factors contributed to 588 extractions, showing male predominance (*p* < 0.05) and significant age-related correlations (*p* < 0.05) (Figure 4). Impacted third molar factors led to 185 extractions, with no significant age-dependent association (*p* > 0.05) (Figure 5).

**Table 2:** Statistical analysis of age distribution differences.

Extraction etiology	<36 years	36–55 years	56–75 years	>75 years	Chi-square (*χ*^2^)	df	*p*-value
Endodontic factors	263	288	388	106	45.71	3	<0.001
Periodontal factors	28	213	297	50	232.80	3	<0.001
Impacted third molar-related factors	53	77	52	3	89.34	3	<0.001
Total	344	578	737	159	427.11	3	<0.001

## 4 Discussion

A total of 1,818 valid cases were included in the study, with an average age of 52.9 years (864 males and 954 females). Endodontic factors were responsible for 1,045 s molar extractions (57.43%), and demonstrated a statistically significant association with age (*p* < 0.05). Periodontal factors contributed to 588 extractions (32.40%), and the association with age was also statistically significant (*p* < 0.05). Impacted third molars accounted for 185 extractions (10.01%), with no significant correlation with age (*p* > 0.05). The distribution of the reasons for mandibular second molar extractions was as follows: endodontic factors (56.68%, *n* = 556), periodontal factors (29.36%, *n* = 288), and impacted third molar factors (13.97%, *n* = 137). For maxillary second molar extractions, the distribution was as follows: endodontic factors (57.43%, *n* = 489), periodontal factors (35.84%, *n* = 300), and impacted third molar factors (5.73%, *n* = 48).

The current investigation revealed the 56–75-year old cohort as the predominant demographic for endodontic disease-related extractions. Elderly patients (>60 years) exhibited increasing extraction rates due to an elevated treatment complexity from heightened dental fragility and root canal calcification, aligning with the finding that advanced age was a significant risk factor for endodontically driven extractions (12, 13). Furthermore, complex root canal anatomies (e.g., C-shaped root canal configurations) and suboptimal prior treatments emerged as critical determinants of ultimate extraction, particularly in molars subjected to high occlusal loads, where structural compromise accelerates irreversible damage progression.

Periodontal disease is the secondary cause leading to the extraction of second molars. Studies indicate that the peak incidence of second molar extraction due to advanced periodontitis occurs in patients aged 50–70 years, with the highest prevalence observed in the 55–65-year-old subgroup (approximately 38.5%), a finding consistent with the chronic progression of alveolar bone resorption and pathological tooth mobility (14–16). Elderly populations (>70 years) exhibited elevated extraction risks, driven by systemic comorbidities (e.g., diabetes mellitus) that accelerate periodontal tissue degradation. Regarding sex disparities, prior research demonstrates a pronounced male predominance (61.2% vs. 38.8%), whereas the current study observed a less marked, but still significant, male predominance (53.8% vs. 46.2%). This divergence may stem from higher smoking prevalence, suboptimal oral hygiene practices, and heightened prostaglandin-mediated inflammatory responses among males (14, 17).

The causes of impacted third molars on adjacent second molars are clinically significant. The present study noted that the mandibular third molar (13.97%) seemed to have a greater tendency to cause adverse effects on the second molar than maxillary third molars (5.73%), and a non-significant male predominance (51.4% vs. 48.6%). Previous epidemiological studies identified the 20–40-year-old group as the high-risk demographic for second molar extractions secondary to third molar impaction, with a peak incidence in 25–35-year-olds (47.3%). However, the current study observed a delayed risk window (36–55 years, 41.8%). This temporal shift aligned with the latent progression of third molar-related pathologies, such as severe distal cervical caries and root resorption, which typically manifest years after the third molar eruption phase (17–25 years) (18). Sex-based analyses historically report male predominance (58.1% vs. 41.9%), potentially attributable to anatomical predispositions (e.g., higher mandibular bone density, mesioangular impaction angles) and delayed treatment-seeking behaviors in males, contrasted with earlier prophylactic interventions in females (19). Mesioangular impactions, characterized by direct mechanical pressure on the distal aspect of second molars, were identified as the primary drivers of irreversible damage through accelerated periodontal pocket formation and localized bone resorption (20). Prophylactic third molar removal effectively mitigated second molar complications, yet delayed management often culminated in structural compromise necessitating extraction.

We suggest that younger populations should prioritize caries prevention strategies (e.g., pit and fissure sealants, routine fluoride varnish applications) and early interventions for incipient endodontic disease. Middle-aged and elderly individuals, especially males, require an intensified focus on periodontal disease management through professional debridement and sustained maintenance therapy. Individuals in early-to-middle adulthood should emphasize third molar eruption monitoring, with prompt intervention (e.g., prophylactic extraction, orthodontic evaluation) upon detecting abnormal third molar positioning to prevent subsequent complications.

This study had several limitations: This retrospective single-center analysis may have been subjected to selection bias, limiting the generalizability of the findings. The results shown by CBCT imaging were relatively objective, but EHR often reflected the dentist's subjective opinions, which may cause interference in this study. Only endings of extraction were collected, regardless of caries, pulpitis, and periapical diseases related to third molars, which might weaken the adverse effects of third molars to second molars. Furthermore, this study failed to carefully consider their systemic factors and adverse behavioral factors. It is imperative to conduct multicenter prospective studies with standardized diagnostic criteria (such as caries progression index, periodontal parameters, impacted tooth classification/depth, etc.). Such studies should incorporate patients' systemic variables and adopt stratified sampling based on factors such as age, sex, and lifestyle to further elucidate the specific risk factors and pathogenic mechanisms leading to the extraction of second molars.

## 5 Conclusion

The main reasons for extracting second molars included endodontic factors, followed by periodontal factors, and impacted third molars. Advanced age was significantly associated with an increased risk of extraction attributable to both endodontic and periodontal disease. Impacted third molar-related factors accounted for a larger proportion of cases of mandibular second molar extraction.

## Data Availability

The original contributions presented in the study are included in the article/Supplementary Material, further inquiries can be directed to the corresponding author.
